# *Staphylococci* in Livestock: Molecular Epidemiology, Antimicrobial Resistance, and Translational Strategies for One Health Protection

**DOI:** 10.3390/vetsci12080757

**Published:** 2025-08-13

**Authors:** Ayman Elbehiry, Eman Marzouk

**Affiliations:** Department of Public Health, College of Applied Medical Sciences, Qassim University, P.O. Box 6666, Buraydah 51452, Saudi Arabia; e.marzouk@qu.edu.sa

**Keywords:** antimicrobial resistance, livestock-associated *Staphylococcus*, one health surveillance, alternative therapeutics, public health

## Abstract

Livestock-associated *Staphylococcus* species—particularly *Staphylococcus aureus* (*S. aureus*), *Staphylococcus pseudintermedius* (*S. pseudintermedius*), and coagulase-negative staphylococci (CoNS)—pose escalating threats to animal health, food safety, and public health due to their evolving antimicrobial resistance (AMR) profiles. This review synthesizes recent insights into the molecular epidemiology, resistance determinants, and host adaptation strategies of these pathogens across food-producing animals. We highlight the role of mobile genetic elements (MGEs), clonal dissemination, and biofilm formation in shaping multidrug resistance (MDR) patterns. Diagnostic advancements, including MALDI-TOF MS, whole-genome sequencing (WGS), and PCR-based assays, are discussed alongside treatment challenges arising from therapeutic failures and limited vaccine efficacy. The review critically examines current AMR surveillance gaps and the need for integrative One Health frameworks that encompass animals, humans, and the environment. Novel tools such as metagenomics, real-time genomic surveillance, and artificial intelligence (AI)-driven analytics are proposed to enhance predictive monitoring and resistance management. Together, these insights underscore the urgency of coordinated, evidence-based interventions to curb the spread of MDR staphylococci and safeguard One Health.

## Simple Summary

*Staphylococcus* species are common bacteria that live on the skin and mucous membranes of livestock. While often harmless, some strains—such as *Staphylococcus aureus* (*S. aureus*), *Staphylococcus pseudintermedius* (*S. pseudintermedius*), and coagulase-negative staphylococci (CoNS)—can cause serious diseases and are increasingly resistant to antibiotics. These pathogens are linked to mastitis in dairy cattle, skin infections in pigs, and wound infections in horses and pets, and they can be transmitted to humans through contact or contaminated food. This review summarizes recent findings on their molecular traits, antibiotic resistance mechanisms, and ability to spread across animals, humans, and the environment. It also discusses new diagnostic tools and alternative treatments such as phage therapy, probiotics, and vaccines. A One Health approach is emphasized to improve surveillance and reduce the risks posed by these multidrug-resistant bacteria.

## 1. Introduction

*Staphylococcus* species comprise a taxonomically diverse group of Gram-positive cocci that ubiquitously colonize the skin, mucosal surfaces, and upper respiratory tract of livestock. While often harmless commensals, several species can transition into opportunistic pathogens in response to stress, immunosuppression, or epithelial disruption. This pathogenic shift is well established for *Staphylococcus aureus* (*S. aureus*), *Staphylococcus pseudintermedius* (*S. pseudintermedius*), *Staphylococcus hyicus* (*S. hyicus*), and *Staphylococcus chromogenes* (*S. chromogenes*), all of which are implicated in mastitis, exudative epidermitis, wound infections, and systemic diseases in cattle, pigs, and small ruminants [1,2,3]. Unlike prior reviews, this article uniquely integrates recent genomic, environmental, and One Health surveillance insights to highlight underexplored reservoirs (e.g., CoNS and environmental niches), translational barriers, and emerging tools such as CRISPR-based functional genomics, artificial intelligence (AI) for antimicrobial resistance (AMR) prediction, and novel phage/probiotic interventions.

Among livestock-associated staphylococci, *S. aureus* is particularly significant due to its global prevalence and major role in bovine mastitis. A recent global meta-analysis estimates that approximately 36% of dairy cattle harbor *S. aureus*, either as a commensal organism or as an intramammary pathogen, contributing to both subclinical and clinical mastitis [4]. In parallel, non-*aureus* staphylococci (NAS)—especially *S. chromogenes*—have emerged as leading causes of subclinical mastitis. Large-scale herd studies report that *S. chromogenes* can account for 30–40% of NAS isolates from intramammary infections, often associated with persistent inflammation and elevated somatic cell counts [5,6]. Although generally considered less virulent than *S. aureus*, NAS infections are frequently chronic, difficult to treat, and may serve as reservoirs for AMR [6]. Collectively, infections caused by *S. aureus* and NAS compromise animal welfare and productivity, leading to economic losses through reduced milk yield, increased veterinary interventions, and early culling.

Bovine mastitis due to *S. aureus* remains one of the most economically damaging diseases in dairy farming. The condition results in significant losses due to reduced milk production, discarded milk, veterinary costs, reproductive inefficiencies, and premature animal removal. In the United States alone, annual losses attributed to mastitis exceed USD 2 billion [7,8]. Globally, the combined impact of clinical and subclinical mastitis is estimated between USD 19.7 and USD 32 billion per year, with low- and middle-income countries disproportionately burdened due to limited access to diagnostics, effective herd management, and veterinary care [9,10,11]. Beyond dairy cattle, *Staphylococcus* species also affect meat-producing animals, where they can cause exudative epidermitis, septic arthritis, and wound infections—frequently resulting in carcass trimming or condemnation and raising food safety concerns [12,13].

Beyond dairy cattle, *Staphylococcus* species also affect pigs, poultry, and horses, where they cause a broad spectrum of infections—including exudative epidermitis in piglets caused by *S. hyicus*, bacterial chondronecrosis and osteomyelitis in poultry linked to *S. aureus* and *S. agnetis*, and dermatitis, wound infections, and septic arthritis in horses. These infections pose significant challenges to animal health and productivity, and may also carry food safety implications [14,15,16,17,18]. In poultry, *S. aureus* and other coagulase-negative staphylococci contribute to lameness, bumblefoot, and cellulitis, impacting production and animal welfare. In horses, colonization with MRSA—particularly CC398 and ST8 lineages—has been increasingly reported in veterinary hospitals, with clinical manifestations ranging from wound infections to osteomyelitis and pneumonia [19,20].

The emergence of AMR *Staphylococcus* strains in livestock—particularly livestock-associated methicillin-resistant *S. aureus* (LA-MRSA)—has intensified public health concerns. Although previously common, the use of antibiotics for prophylaxis and growth promotion in livestock has been significantly curtailed in many developed countries due to stringent regulatory reforms. In the European Union, Regulation (EU) 2019/6—effective from 28 January 2022—explicitly prohibits the use of antimicrobials for growth promotion and restricts prophylactic use to exceptional cases in individual animals, while metaphylactic use is permitted only under strict veterinary oversight [21]. LA-MRSA lineages, notably clonal complex 398 (CC398) and ST9, have become endemic in pig and poultry operations across Europe, Asia, and North America [22,23,24,25]. These strains are not restricted to animals; they are increasingly detected in humans with occupational exposure, including farmers, veterinarians, and abattoir workers. Colonization rates with LA-MRSA, particularly CC398, can reach 77–86% among such individuals. While many colonized people remain asymptomatic, documented clinical manifestations include skin and soft tissue infections, pneumonia, bloodstream infections, and surgical site infections [26,27].

Zoonotic transmission of *Staphylococcus* species extends beyond direct contact with livestock. Contaminated animal-derived products—such as raw milk, meat, and cheese—can serve as vehicles for toxigenic and resistant *S. aureus*, contributing to foodborne outbreaks of staphylococcal enterotoxicosis [28,29,30]. Raw milk and other unpasteurized dairy products have been increasingly recognized as reservoirs of antimicrobial resistance genes (ARGs), harboring a broader and more abundant resistome compared to pasteurized counterparts. This elevated ARG burden is largely attributed to the presence of resistant *Staphylococcus* spp. and other bacteria shed from infected udders, as well as environmental contaminants introduced during milking, storage, and handling. Consumption of such products—especially by immunocompromised individuals—may facilitate horizontal gene transfer of ARGs to the human gut microbiota via mobile genetic elements, thereby contributing to the spread of antimicrobial resistance [31,32].

In addition, environmental contamination through farm dust, wastewater, and manure-amended soils has been shown to harbor resistant strains, underscoring the role of livestock farms as ecological amplifiers of AMR bacteria [33,34]. The bidirectional nature of transmission between animals and humans further complicates infection control, particularly in production systems lacking adequate biosecurity measures.

The pathogenicity of *Staphylococcus* species is underpinned by their extraordinary genomic flexibility. These bacteria routinely acquire and disseminate mobile genetic elements (MGEs)—including plasmids, transposons, prophages, and staphylococcal pathogenicity islands (SaPIs)—which encode key AMR genes (e.g., *mecA*, *mecC*, *tetM*, *ermB*) and virulence factors (e.g., enterotoxins, leukocidins, MSCRAMMs) [35,36]. SaPIs and bacteriophages play a pivotal role in horizontal gene transfer, facilitating the spread of resistance and virulence determinants across diverse staphylococcal populations [37]. In addition to phage-mediated transfer, ARG dissemination in *Staphylococcus* spp. commonly occurs via plasmids, transposons, and staphylococcal chromosomal cassettes (e.g., SCCmec), which facilitate horizontal gene transfer across strains and species. Genomic studies have confirmed that LA-MRSA lineages such as CC398 share multiple MGEs with human-adapted strains, reinforcing their zoonotic potential and evolutionary adaptability [12].

Moreover, species such as *S. pseudintermedius* and *S. hyicus*—previously considered host-restricted—are now increasingly associated with opportunistic infections in humans. These include skin and soft tissue infections and bloodstream infections, particularly in pet owners, immunocompromised individuals, and others with close animal contact [38,39]. These findings reflect the dynamic role of MGEs in shaping staphylococcal evolution and highlight the need for integrated surveillance and control strategies.

**Figure 1:**
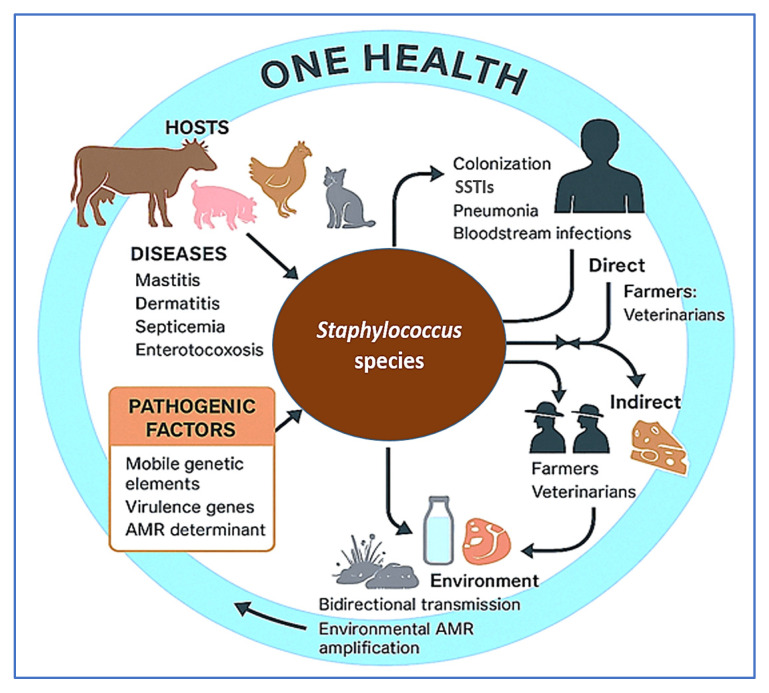
Epidemiological and genomic framework of livestock-associated *Staphylococcus* species within a One Health context. This conceptual diagram illustrates the interplay between animal hosts, pathogenic traits, and routes of zoonotic transmission. Major livestock species act as reservoirs for pathogenic and antimicrobial-resistant *Staphylococcus* species, which cause a range of clinical diseases. Human exposure occurs through direct contact (farmers, veterinarians) or indirectly via contaminated animal products and environmental reservoirs. Pathogenicity is driven by mobile genetic elements, virulence genes, and antimicrobial resistance (AMR) determinants. The bidirectional transmission and environmental amplification of resistance genes underscore the need for integrated One Health surveillance and stewardship. This figure was created using a combination of BioRender.com (via a private academic free trial account) and Microsoft PowerPoint (Microsoft 365), both of which permit use in open-access scientific publications.

Given the growing significance of staphylococci at the interface of animal health, food safety, and public health, a comprehensive, integrative approach is warranted. This review aims to synthesize current knowledge on livestock-associated *Staphylococcus* spp., focusing on their taxonomy, genomic diversity, virulence mechanisms, and AMR profiles. It further explores zoonotic risks, advances in diagnostic and surveillance tools, and emerging prevention and control strategies—including vaccines, probiotics, phage therapy, and antimicrobial stewardship. Finally, we highlight key research gaps and future directions within a One Health framework that acknowledges the interconnectedness of human, animal, and environmental health in addressing the multifaceted challenges posed by staphylococcal infections. To visually synthesize the multifaceted interplay between *Staphylococcus* spp., livestock, human exposure, and environmental risk, Figure 1 illustrates the core epidemiological and genomic relationships within a One Health framework. This diagram contextualizes the zoonotic transmission dynamics, AMR factors, and disease burden relevant to both animal and public health.

## 2. Taxonomic and Genomic Diversity of Livestock *Staphylococcus* spp.

Livestock-associated *Staphylococcus* species constitute a phylogenetically diverse and genomically adaptable group of Gram-positive bacteria. Among more than 50 recognized species, the ones most frequently associated with animal disease include *S. aureus*, *S. pseudintermedius*, *S. hyicus*, and *S. chromogenes*; others like *S. sciuri* and *S. simulans* are occasionally implicated and increasingly recognized as reservoirs of AMR [13,39,40]. Their evolutionary success stems in part from remarkable genomic plasticity mediated by MGEs —such as plasmids, prophages, transposons, SaPIs, and staphylococcal cassette chromosome mec (SCCmec) cassettes—which enable horizontal gene transfer of antibiotic resistance determinants (*mecA*, *mecC*, *tetM*, *ermB*) and virulence factors like enterotoxins, exfoliative toxins, leukocidins, and MSCRAMMs [35,36,41].

### 2.1. Staphylococcus aureus (S. aureus)

*S. aureus* is the most extensively studied staphylococcal species in both human and veterinary contexts, with numerous host-adapted lineages causing disease in livestock. In cattle, particularly dairy herds, bovine-adapted clonal complexes (CCs) such as CC97, CC151, and CC133 predominate and are closely associated with subclinical and clinical mastitis. These lineages are phylogenetically distinct from human-adapted lineages like CC5 and CC8, suggesting long-term host specialization [42,43,44]. Comparative genomic analyses have shown that bovine isolates commonly harbor specific surface adhesin genes, such as *clfB*, *fnbB*, and *cna*, which facilitate adherence to mammary epithelial cells [45,46]. Additionally, genes related to lactose metabolism and metal ion acquisition (particularly iron and manganese) are consistently enriched in bovine *S. aureus* isolates, reflecting metabolic adaptation to growth in milk and the mammary gland environment [47]. In contrast, bovine *S. aureus* isolates frequently lack the human immune evasion cluster (IEC) genes—*scn*, *chp*, and staphylokinase (*sak*)—suggesting host-specific genome adaptation; these genes are notably absent or rare in animal isolates compared to their high prevalence (up to 90%) in human strains [48].

During persistent intramammary infections, *S. aureus* undergoes significant microevolution. Longitudinal genome studies have documented within-host diversification including point mutations, insertions/deletions, and regulatory changes—particularly affecting the *sigB* stress-response regulon and capsule biosynthesis genes [49,50]. These genetic alterations often result in the emergence of small-colony variants (SCVs), a phenotype linked to enhanced biofilm formation, reduced metabolic activity, and increased intracellular persistence—traits known to impede treatment efficacy and promote chronic infection [51,52]. WGS of bovine *S. aureus* isolates reveals a highly dynamic accessory genome enriched with MGEs, including prophages, SaPIs, plasmids, and resistance cassettes. These MGEs often encode virulence factors such as enterotoxins, leukocidins, and immune-modulating proteins, with distribution patterns varying by clonal lineage and geography [53].

A large-scale WGS study of bovine milk isolates from Canada revealed significant accessory genome variability, with distinct virulence gene profiles and sporadic presence of AMR determinants, including *blaZ* and *tetK* [42]. Comparative genomic analyses of mastitis-associated *S. aureus* isolates from India (n = 41) revealed an open pan-genome enriched with mobile genetic elements, including prophages, plasmids, and AMR genes, underscoring ongoing horizontal gene transfer and adaptive evolution within dairy environments [54]. Additionally, European bovine isolates frequently harbor the leukocidin LukMF’, encoded by prophages, a toxin highly cytotoxic to bovine neutrophils and associated with severe mastitis cases [55]. These findings highlight the remarkable genomic plasticity and evolutionary adaptability of *S. aureus*, enabling persistence within the bovine host, immune evasion, and occasional interspecies transmission. Given its central role in bovine mastitis and zoonotic potential, *S. aureus* remains a priority for genomic surveillance, innovative diagnostics, and next-generation vaccine development to safeguard livestock health.

### 2.2. Staphylococcus pseudintermedius (S. pseudintermedius)

Although *S. pseudintermedius* is primarily recognized as a commensal and opportunistic pathogen in dogs, increasing evidence points to its presence in livestock, especially in swine and small ruminants. MDR clones—particularly sequence types ST71 and ST45—have been reported in both companion animals and livestock across Europe, Asia, and the Americas [56,57]. These strains typically harbor the SCCmec element, conferring methicillin resistance, and exhibit co-resistance to fluoroquinolones, macrolides, tetracyclines, and aminoglycosides [13,58]. The virulence of *S. pseudintermedius* is driven by a diverse suite of pathogenic determinants, including leukocidins (*lukF/S-I*), exfoliative toxins (e.g., *expA*, *expB*, *siet*), and cell-wall–anchored Sps proteins that facilitate tissue colonization, immune evasion, and inflammation [59,60]. These genes are often located on prophages and other mobile genetic elements, highlighting the role of horizontal gene transfer in shaping *S. pseudintermedius* pathogenicity [60,61]. Notably, methicillin-resistant *S. pseudintermedius* (MRSP) strains typically possess larger and more complex accessory genomes than methicillin-susceptible counterparts, driven by the acquisition of resistance islands and prophage integrations [59,62].

One of the key virulence traits of *S. pseudintermedius* is its robust ability to form biofilms, which contribute significantly to chronic infections and therapeutic failure. The *icaABCD* operon, essential for the synthesis of polysaccharide intercellular adhesin, is widely distributed among clinical isolates and facilitates biofilm development on mucosal and epithelial surfaces [63,64]. A majority of strains—including both methicillin-susceptible and MRSP clones—are moderate to strong biofilm producers, underscoring the critical role of biofilms in disease persistence [13,65]. ST71, a globally disseminated MRSP lineage, exhibits enhanced biofilm formation and increased adhesion to canine keratinocytes, facilitating prolonged colonization and antimicrobial resistance [39,66]. These clones typically harbor the SCCmec element along with multiple resistance genes targeting β-lactams, tetracyclines, aminoglycosides, and fluoroquinolones [67,68].

Zoonotic transmission of *S. pseudintermedius* is increasingly documented among pet owners, veterinarians, and livestock handlers. Although human infections are relatively uncommon, reported cases include wound infections, bacteremia, endocarditis, and prosthetic joint infections. Molecular studies confirm that many human isolates are genetically indistinguishable from canine MRSP strains. For example, Somayaji et al. [69] identified identical genotypes in clinical cases involving close contact with dogs in Canada, while Lozano et al. [70] reported matching pulsed-field gel electrophoresis patterns and multilocus sequence types between human and canine isolates in Spain. These findings underscore the public health implications of MRSP, especially given its rising multidrug resistance and genomic plasticity. As *S. pseudintermedius* continues to expand its host range and adapt to new ecological niches, there is an urgent need for integrated One Health surveillance, advanced molecular diagnostics, and targeted antimicrobial stewardship to mitigate its clinical and zoonotic impact.

### 2.3. Staphylocccus hyicus (S. hyicus) and Staphylococcus chromogenes (S. chromogenes)

Although relatively understudied compared to *S. aureus*, *S. hyicus* and *S. chromogens* are important veterinary pathogens with distinct host associations and virulence profiles. *S. hyicus* is primarily known for causing exudative epidermitis in piglets—a severe dermatological condition characterized by widespread greasy skin lesions, dehydration, and high mortality rates [71]. The pathogenicity of *S. hyicus* is largely driven by its production of exfoliative toxins—*ExhA*, *ExhB*, *ExhC*, and *ExhD*—which are serine proteases targeting porcine desmoglein-1, resulting in epidermal cell detachment and compromised skin barrier integrity [72,73]. These toxin genes demonstrate variable genomic localization: *exhA*, *exhC*, and *exhD* are typically situated on the bacterial chromosome, while *exhB* is often plasmid-encoded—facilitating ease of horizontal gene transfer and contributing to strain heterogeneity [72,74]. In addition to toxic factors, *S. hyicus* from swine frequently carries resistance determinants located on plasmids and chromosomes—including genes conferring resistance to β-lactams, tetracyclines, macrolides, lincosamides, and pleuromutilins—contributing to treatment failures in pig production systems [75].

In contrast, *S. chromogenes* is a coagulase-variable non-aureus staphylococcal species that plays a predominant role in subclinical bovine mastitis, particularly in heifers and primiparous cows. MLST-based studies have identified significant clonal diversity among isolates, yet certain sequence types—such as ST1 and ST6—are repeatedly associated with intramammary infections on specific farms, indicating adaptation and persistence within the bovine udder [76,77]. Genomic analyses further reveal that *S. chromogenes* strains encode surface adhesins, biofilm-associated proteins, and multiple iron-acquisition systems, including siderophore transporters and heme-binding proteins, which facilitate persistent colonization in the iron-restricted environment of the mammary gland [76,77]. Although typically considered less virulent than *S. aureus*, recent sequencing efforts have uncovered AMR genes and potential toxin-encoding regions in *S. chromogenes*, suggesting that some strains may pose a more significant risk than previously appreciated [76].

These findings underscore the evolutionary adaptation of *S. hyicus* and *S. chromogenes* to their respective swine and bovine hosts. *S. hyicus* utilizes exfoliative toxins and plasmid-mediated traits to colonize porcine skin, while *S. chromogenes* persists in the bovine udder via adhesins, biofilm formation, and iron acquisition systems. Both species increasingly harbor AMR genes, complicating treatment. Their capacity to persist, evade immune responses, and adapt genomically highlights their veterinary importance. Effective control requires enhanced genomic surveillance, improved hygiene, autogenous vaccines, and prudent antimicrobial use to mitigate their impact on animal health and productivity.

### 2.4. Host Adaptation and the MGEs

The success of livestock-associated *S. aureus*, particularly CC398, as a pathogen is largely attributed to its remarkable genomic adaptability and host-specific evolution. Originally a human-associated lineage, CC398 underwent a host jump to livestock—especially pigs, cattle, and poultry—facilitated by the loss of human immune evasion genes (e.g., *scn*, *chp*) and the acquisition of MGEs tailored to animal hosts [78]. Key MGEs such as SCCmec type V, Tn916, prophages, and SaPIs have been identified in livestock strains, frequently encoding resistance to tetracyclines, macrolides, and methicillin, as well as virulence factors including leukocidins and enterotoxins [13,79]. Antimicrobial selection pressures in agricultural settings play a central role in shaping the accessory genome of *S. aureus*. For instance, extensive use of tetracyclines in swine farming has driven the expansion of CC398 strains harboring *tetM*, *ermC*, and other resistance determinants on plasmids and transposons [80]. Genomic studies have further revealed recurrent deletions in capsule biosynthesis loci (e.g., cap5, cap8)—a trait associated with immune evasion—and the gain of metabolic genes such as lactose operons (*lacA*, *lacB*) that enable growth in milk-rich environments like the bovine udder [46,81].

**Figure 2:**
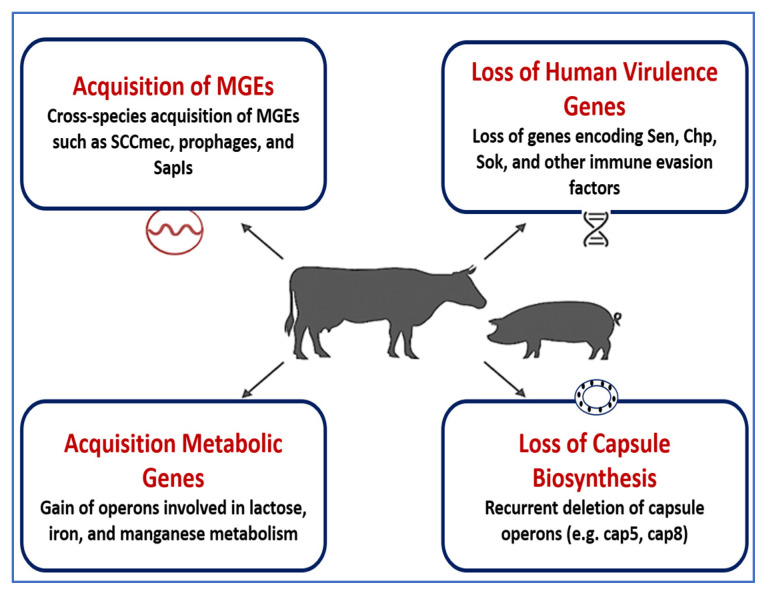
Genomic mechanisms facilitating host adaptation in livestock-associated *Staphylococcus* species. This schematic illustrates the key genomic adaptations that enable *Staphylococcus* species to persist and specialize in livestock hosts. These include: (1) Acquisition of mobile genetic elements (MGEs) such as the staphylococcal cassette chromosome mec (SCCmec), prophages, and staphylococcal pathogenicity islands (SaPIs), which facilitate horizontal gene transfer; (2) Loss of human-associated virulence genes, including staphylococcal complement inhibitor (SCIN), chemotaxis inhibitory protein (CHIPS), and other immune evasion factors, reducing human tropism; (3) Acquisition of metabolic operons for lactose, iron, and manganese utilization, enhancing adaptation to the nutrient profile of animal hosts; and (4) Loss of capsule biosynthesis genes, such as cap5 and cap8, which may modulate immune recognition and biofilm formation. Together, these events promote niche specialization, immune evasion, and persistence in livestock environments, reinforcing the evolutionary divergence between human- and animal-associated *Staphylococcus* lineages. This figure was created using Microsoft PowerPoint (Microsoft 365), which is permitted for use in open-access scientific publications.

Recent comparative genomics have shown that these adaptive processes are not random but follow predictable evolutionary patterns, suggesting strong selective constraints within the livestock niche. Notably, livestock MRSA isolates can retain zoonotic potential, as spillover events into humans—particularly farmers, abattoir workers, and veterinarians—have been well documented [2,82,83]. These findings reinforce the dynamic nature of MGEs in driving host specificity, resistance, and interspecies transmission. To mitigate the public health risks posed by these adaptive staphylococcal lineages, it is crucial to implement integrated One Health genomic surveillance programs. These efforts should monitor the emergence of novel MGEs, track horizontal gene transfer across reservoirs, and inform antimicrobial stewardship across veterinary and human sectors. Figure 2 summarizes the genomic mechanisms underlying host adaptation in livestock-associated *Staphylococcus* spp., emphasizing the role of MGEs, regulatory evolution, and metabolic specialization in shaping strain ecology and zoonotic potential. Table 1 summarizes the major genomic mechanisms underpinning host adaptation in livestock-associated *Staphylococcus* spp., highlighting species-specific strategies such as immune evasion, metabolic adaptation, toxin production, and AMR. These adaptations, largely mediated by MGEs and accessory genome expansions, facilitate persistent colonization, zoonotic transmission, and treatment failure in veterinary contexts.

## 3. Virulence Factors and Pathogenesis

The pathogenicity of livestock-associated *Staphylococcus* species is driven by a highly coordinated array of virulence factors that enable host colonization, immune evasion, and long-term persistence. Adhesion to host tissues is mediated by microbial surface components recognizing adhesive matrix molecules (MSCRAMMs), including *ClfB*, *FnbB*, and *Cna*, which facilitate tissue-specific binding, particularly within the udder and skin [45,84]. Following colonization, the secretion of potent toxins—such as LukMF′, exfoliative toxins, and hemolysins—disrupts epithelial integrity and modulates host immune responses [72,85]. Biofilm formation, orchestrated by the *icaADBC* operon and modulated by global regulators like *agr* (accessory gene regulator) and *sarA*, confers antimicrobial tolerance and facilitates chronic infection [52,63]. Immune evasion is further enhanced by capsular polysaccharides, the expression of protein A, and prophage-encoded factors such as chemotaxis inhibitory protein of *Staphylococcus* (CHIPS), staphylococcal complement inhibitor (SCIN), and SAK [48]. Many of these determinants are encoded on MGEs—including plasmids, prophages, transposons, and SaPIs—which mediate horizontal gene transfer and rapid adaptation to diverse ecological niches [36,78].

Virulence traits vary substantially across species and host animals: *S. aureus* adapts for growth in milk; *S. hyicus* produces porcine-specific exfoliative toxins; *S. pseudintermedius* forms robust biofilms on canine skin [46,59,71]. These host-specific adaptations reflect the remarkable genomic plasticity of *Staphylococcus* spp. and underscore the need for genomic surveillance and precision control strategies. The following subsections provide a mechanistic overview of five core virulence strategies: adhesion, toxin production, biofilm development, immune evasion, and host-adaptive variability.

### 3.1. Surface Adhesins and Host Tissue Tropism

A critical first step in *Staphylococcus* pathogenesis is adhesion to host tissues, primarily mediated by MSCRAMMs. These surface-anchored proteins enable tight binding to extracellular matrix components such as fibrinogen, fibronectin, collagen, and cytokeratin, facilitating colonization of epithelial barriers and connective tissues. Well-characterized adhesins include clumping factors *ClfA* and *ClfB*, fibronectin-binding proteins *FnbA* and *FnbB*, and collagen-binding adhesin *Cna*. These are especially prominent in *S. aureus* strains from dairy cattle, where they enhance adherence to bovine mammary epithelial and stromal cells [45,84].

*ClfB* binds cytokeratin 10 and loricrin, aiding in nasal colonization and intramammary persistence. Meanwhile, *FnbA* and *FnbB* promote cellular invasion by triggering integrin-mediated endocytosis [44,89]. In clinical bovine mastitis, enrichment of *clfB* and *fnbB* correlates with more severe and persistent infections. Similarly, *S. chromogenes*, a dominant NAS species in subclinical mastitis, encodes iron-regulated and LPXTG-motif adhesins that promote survival in the iron-restricted, lipid-rich udder environment [76]. Genomic analyses show clonal enrichment of these adhesion and biofilm-related genes in intramammary isolates.

Adhesion is both species- and host-specific. Ruminant-adapted *S. aureus* strains show distinct *clfA*, *clfB*, *fnbA*, and *ebpS* profiles, along with unique *sas* family adhesins that are uncommon in human strains [43,53]. These adaptations reflect host-specific selective pressures and extend to associated MGEs and restriction-modification systems. Collectively, MSCRAMM expression enables *Staphylococcus* spp. to establish persistent colonization, resist immune clearance, and maintain chronic infections. Understanding these adhesion mechanisms is essential for designing targeted interventions such as anti-adhesion vaccines, competitive exclusion strategies, and species-specific diagnostics for livestock pathogens.

### 3.2. Exotoxins and Enzymatic Factors

Exotoxins are central to the virulence of livestock-associated *Staphylococcus* species, contributing to epithelial damage, immune evasion, and disease dissemination. In bovine-adapted *S. aureus*, the leukotoxin LukMF′—encoded by prophages—is a major virulence determinant with potent cytotoxicity against bovine neutrophils. It is the most abundantly secreted leukotoxin in bovine isolates and is strongly associated with the severity of clinical mastitis, underscoring its pivotal role in pathogenesis [85]. In addition to LukMF′, *S. aureus* produces a suite of cytolytic toxins, including α-, β-, and γ-hemolysins and phenol-soluble modulins, which disrupt host cell membranes, promote inflammation, and facilitate biofilm dispersal. These toxins collectively damage host tissues, facilitate immune subversion, and contribute to systemic spread.

In pigs, *S. hyicus* secretes exfoliative toxins (ExhA–D), which are serine proteases that target porcine desmoglein-1, disrupting epidermal cohesion and causing exudative epidermitis (“greasy pig disease”). These toxins not only cause epithelial detachment but also induce strong proinflammatory responses (e.g., IL-1β, IL-8, IL-12), amplifying tissue damage and systemic effects [72,90].

Zoonotic strains of *S. aureus* also produce classical staphylococcal enterotoxins and newer variants (SEG–SEI), which act as heat-stable superantigens. These toxins, commonly found in raw milk and dairy products, retain biological activity post-pasteurization and are responsible for staphylococcal food poisoning outbreaks. A systematic review of 24 studies estimated that ~11.6% of raw milk samples harbor enterotoxigenic *S. aureus*, highlighting the importance of rigorous dairy hygiene and food safety practices [91]. The diversity of exotoxins and enzymatic factors reflects the species’ ability to adapt to different hosts and niches. These virulence agents not only cause acute disease but also promote chronicity and transmission. As such, they represent prime targets for next-generation therapeutics, including toxoid-based vaccines, neutralizing antibodies, and phage-derived anti-toxin agents.

### 3.3. Biofilm Formation and Chronicity

Biofilm formation is a key virulence mechanism in livestock-associated *Staphylococcus* species, promoting chronic infection, antimicrobial tolerance, and evasion of host immunity. This complex process is primarily driven by the icaADBC operon, which encodes enzymes responsible for synthesizing polysaccharide intercellular adhesin (PIA)—a crucial structural component of the extracellular matrix. PIA enhances bacterial adherence to surfaces, facilitates intercellular aggregation, and provides protection from environmental and immune stressors. High biofilm-forming capabilities have been reported in *S. aureus* and *S. pseudintermedius* strains isolated from bovine mastitis, canine pyoderma, and medical device-associated infections in veterinary practice [64,92]. These biofilms enable bacteria to persist under adverse conditions such as nutrient limitation, antimicrobial exposure, and immune surveillance, particularly in the udder, skin, and surgical sites.

Two key global regulators—accessory gene regulator (agr) and staphylococcal accessory regulator (*sarA*)—play opposing roles in biofilm modulation. The agr system, which governs quorum sensing, promotes the expression of dispersal factors and suppresses adhesins, thus negatively regulating mature biofilm formation. In contrast, *sarA* enhances biofilm stability by repressing extracellular protease and nuclease activity, thereby preserving matrix integrity. Disruption of *sarA* function results in impaired biofilm architecture and increased matrix degradation [93].

Biofilm-associated cells exhibit profound physiological shifts, including reduced metabolic activity, altered membrane charges, and emergence of SCVs—a phenotype associated with persistence and antibiotic tolerance. These changes contribute to tolerance rather than classical resistance, making infections difficult to eradicate even with prolonged antibiotic therapy. This is especially problematic in persistent intramammary infections, surgical implants, and indwelling devices [94,95]. Given its contribution to persistence and treatment failure, biofilm formation is increasingly being targeted in the development of novel therapeutics. Promising approaches include quorum-sensing inhibitors, enzymatic dispersal agents, and anti-adhesion molecules, which aim to disrupt matrix structure, inhibit formation, or enhance antibiotic penetration. These strategies are critical to improving clinical outcomes and preventing relapses in veterinary infections.

### 3.4. Immune Evasion Strategies

Livestock-associated *Staphylococcus* species have evolved a sophisticated arsenal of immune evasion mechanisms that enable persistent colonization, recurrent infection, and evasion of host defenses [96]. One of the most fundamental strategies involves capsular polysaccharides—primarily types 5 and 8 in *S. aureus*—which inhibit opsonophagocytosis and impair complement-mediated killing by masking surface antigens [97]. Another hallmark immune evasion mechanism is the expression of staphylococcal protein A (SpA), which binds to the Fc region of immunoglobulin G (IgG). This interaction blocks opsonization and disrupts antibody-mediated phagocytosis [98]. Moreover, SpA can bind to the Fab region of B-cell receptors, impairing adaptive immune responses. Preclinical studies have shown that a genetically detoxified SpA variant (SpA*) can elicit strong Th1-skewed antibody responses when combined with the leukotoxin LukAB, supporting its inclusion in multicomponent vaccines [99]. These findings support the feasibility of targeting immune evasion proteins such as SpA for next-generation vaccines in both veterinary and zoonotic contexts.

Additional immune evasion is mediated by a suite of secreted virulence factors encoded by the often carried on β-converting bacteriophages [100]. These include the CHIPS, which blocks neutrophil chemotaxis by binding formyl peptide and C5a receptors; the staphylococcal complement inhibitor (SCIN), which impairs complement activation by stabilizing C3 convertases; and SAK, which activates host plasminogen to degrade opsonins such as C3b and IgG. These factors are often encoded by the φSa3 family of β-converting prophages, and their presence potentiates early innate immune evasion and systemic dissemination [101]. However, the distribution of these factors varies markedly among *Staphylococcus* lineages. In particular, the IEC, typically carried on φSa3 prophages, is notably absent in livestock-associated MRSA CC398 isolates—reflecting a loss of human-specific immune modulators and an adaptation to animal hosts [102]. This absence underscores the host-specific selection pressures shaping the virulence repertoire and zoonotic potential of staphylococcal lineages.

Emerging evidence highlights that immune evasion is not limited to *S. aureus*, but is also a critical feature in other veterinary-relevant species. *S. pseudintermedius*, a leading cause of canine pyoderma and post-surgical infections, expresses SpsQ—a cell wall–anchored homolog of staphylococcal protein A—that binds the Fc region of canine IgG, thereby impairing opsonization and phagocytic clearance. This protein is strongly expressed among dominant clonal lineages such as ST68 and ST71 and has been directly linked to enhanced serum survival and immune escape in canine hosts [103].

Moreover, *S. pseudintermedius* produces staphylococcal binder of immunoglobulin variants that interact with complement components C3 and factor H, further inhibiting complement-mediated opsonophagocytosis [104]. These immune evasion strategies—including structural defenses and secreted modulators—form a coordinated mechanism that enables *Staphylococcus* spp. to persist within animal hosts despite immune activation. Elucidating these pathways is vital for informing the development of next-generation vaccines and therapeutic strategies that target conserved virulence determinants across species.

### 3.5. Interspecies and Intraspecies Variability

Comparative genomic studies have underscored the remarkable heterogeneity in virulence and resistance determinants among *Staphylococcus* species, as well as within individual clonal complexes [105,106,107]. This genomic plasticity is a major driver of host adaptation, pathogenicity, and epidemiological success across diverse ecological niches. In *S. aureus*, lineages associated with ruminants—particularly those causing bovine mastitis—typically lack human-specific immune evasion genes (e.g., *scn*, *chp*, *sak*) but carry lineage-specific adhesins and metabolic adaptations, notably lactose utilization operons, which enhance fitness and survival in milk-rich environments. Notably, these strains frequently encode lactose metabolism operons and milk-specific nutrient acquisition systems that enhance survival and growth in the mammary gland. Recent CRISPRi-seq analyses revealed that genes related to lactose uptake, iron acquisition, and oxidative stress response are essential for intramammary fitness, highlighting selective pressures imposed by the milk microenvironment [108].

Conversely, *S. pseudintermedius*, a dominant commensal and opportunistic pathogen in dogs, exhibits a virulence profile characterized by exfoliative toxins, Panton–Valentine-like leukocidins (lukS/F-I), and robust biofilm-forming capabilities. These virulence traits are often encoded on MGEs and are frequently associated with ARGs, particularly in epidemic clones such as ST68, ST71, and ST258. WGS studies have demonstrated that MRSP lineages co-harbor resistance to β-lactams, aminoglycosides, fluoroquinolones, and macrolides—posing significant challenges to treatment in veterinary settings [57,109].

Even within a single species, considerable intraspecific variability exists, reflecting host adaptation, ecological pressures, and genome plasticity. In *S. aureus*, human-associated clonal complexes such as CC5 and CC8 commonly harbor IEC genes and staphylococcal enterotoxin clusters. In contrast, livestock-associated lineages like CC398 have lost the IEC—typically encoded on φSa3 prophages—but have acquired genes linked to biofilm formation, environmental stress resistance, and epithelial adherence, facilitating their adaptation to ruminant hosts [101]. Similarly, *S. pseudintermedius* exhibits substantial genomic heterogeneity across geographic regions and clonal lineages. Recent population-level analyses have demonstrated marked variability in capsule types, surface protein alleles, and toxin gene content, all of which correlate with specific host associations, antimicrobial usage patterns, and local epidemiological pressures [110].

This high degree of intraspecific diversity—shaped by horizontal gene transfer, recombination events, and mobile genetic elements—poses significant barriers to the development of broad-spectrum vaccines or universal diagnostics. As such, precision approaches are required for effective staphylococcal control, emphasizing the integration of molecular epidemiology, genomics, and host–pathogen interaction studies. Tailored interventions that consider the unique genetic and ecological profiles of staphylococcal populations will be essential for advancing veterinary infectious disease management and mitigating the zoonotic potential of these pathogens.

### 3.6. Role of MGEs in Virulence and Adaptation

MGEs play a pivotal role in shaping the virulence, host specificity, and ecological versatility of livestock-associated *Staphylococcus* species. These elements—including plasmids, prophages, insertion sequences, transposons, SaPIs, and integrative conjugative elements—act as key mediators of horizontal gene transfer. Through this mechanism, MGEs facilitate the dissemination of genes associated with virulence, AMR, metabolic flexibility, and immune evasion across strains and species boundaries [111,112]. Among these, the φSa3 family of β-converting prophages is one of the most studied. These elements integrate into the *hlb* gene and often carry the IEC, including genes such as *scn*, *chp*, *sak*, and occasionally *sea* [112]. While φSa3 is commonly present in human-adapted *S. aureus* strains, it is frequently absent in livestock-associated lineages like MRSA CC398, reflecting a shift away from human-specific immune modulators toward animal host adaptation [78].

Another important class of MGEs, the SaPIs, frequently encodes superantigens (e.g., *seb*, *sec*, *tsst-1*), exfoliative toxins (*eta*, *etb*), and enterotoxins (*seg*, *sei*), contributing to toxin-mediated diseases in both humans and animals [113]. SaPIs are typically mobilized by helper bacteriophages, enabling efficient transfer across *Staphylococcus* strains and occasionally into other Gram-positive genera. In *S. hyicus*, for example, exfoliative toxin genes such as *exhA–D*—key contributors to porcine exudative epidermitis—are often located on SaPI-like chromosomal islands or prophage-associated cassettes, supporting the hypothesis of horizontal acquisition within porcine lineages [72]. Plasmids also serve as major vectors of virulence and resistance. In both *S. aureus* and *S. pseudintermedius*, plasmids commonly harbor genes encoding staphylococcal enterotoxins (e.g., *sed*, *sej*), adhesion factors, and AMR determinants such as *tetK*, *ermC*, and *blaZ* [67,114]. Co-localization of these virulence and resistance genes enhances bacterial survival under selective pressures prevalent in livestock environments.

ICEs contribute further to gene flux by mediating large-scale chromosomal transfers. In *S. aureus*, ICEs have been shown to carry genes for heavy metal resistance, toxin–antitoxin systems, and restriction–modification machinery, which collectively enhance resilience in diverse farm-associated ecological niches [115]. The ability of MGEs to rapidly restructure the genome significantly increases the adaptability of *Staphylococcus* species. Their role in cross-species transmission—especially in agricultural environments with intense antimicrobial use—highlights the urgent need for molecular surveillance programs. Recent evidence advocates for phage-resolved genomics and MGE profiling as tools to monitor the emergence and dissemination of mobile virulence islands, particularly in zoonotic and veterinary pathogens [116].

In summary, MGEs are central to the evolutionary success of livestock-associated *Staphylococcus* spp., equipping them with dynamic and transferable arsenals of virulence and resistance. Their involvement in host adaptation, ecological persistence, and interspecies spread emphasizes the importance of integrating MGE analysis into routine genomic epidemiology. This approach can provide critical insights into the zoonotic potential and evolutionary trajectories of emergent staphylococcal strains, informing proactive control measures and One Health-based interventions.

**Figure 3:**
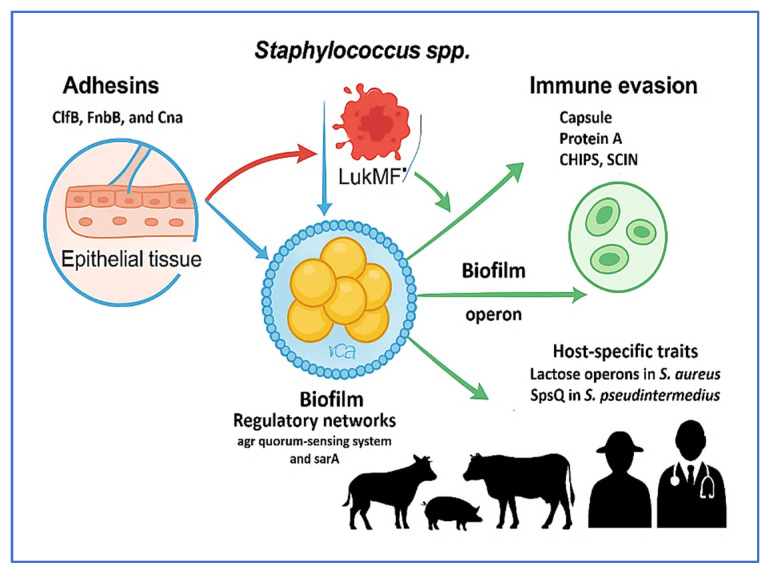
Integrated virulence mechanisms of livestock-associated *Staphylococcus* species. This figure illustrates key virulence strategies used by *Staphylococcus aureus* and *Staphylococcus pseudintermedius* to support colonization, persistence, and zoonotic transmission. These include: (1) Adhesion via microbial surface components recognizing adhesive matrix molecules (MSCRAMMs), including *clfB*, *fnbB*, and *cna*; (2) Cytotoxin production, including *lukMF′* and *exhA–D*, promoting epithelial injury and immune disruption; (3) Biofilm formation through the *icaADBC* operon and regulators *agr* and *sarA*, facilitating environmental persistence and treatment resistance; (4) Immune evasion mediated by capsular polysaccharides, staphylococcal protein A (SpA), chemotaxis inhibitory protein of *Staphylococcus aureus* (CHIPS), staphylococcal complement inhibitor (SCIN), and staphylokinase (SAK); (5) Host-specific adaptations involving genes such as lactose operons and *spsQ*, which enhance survival in animal hosts. These mechanisms collectively promote the success of livestock-associated *Staphylococcus* spp. and underscore the need for integrated One Health approaches in surveillance and control. This figure was created using Microsoft PowerPoint (Microsoft 365), which is permitted for use in open-access scientific publications.

Figure 3 illustrates the multifactorial virulence mechanisms employed by livestock-associated *Staphylococcus* spp., including *S. aureus* and *S. pseudintermedius*. The diagram summarizes five core pathogenic strategies: adhesion via MSCRAMMs (*ClfB*, *FnbB*, *Cna*), cytotoxin secretion (LukMF′, ExhA–D), biofilm formation (*icaADBC*, *agr*, *sarA*), immune evasion (capsule, Protein A, CHIPS, SCIN, SAK), and host-specific adaptations (e.g., lactose operons in *S. aureus*, SpsQ in *S. pseudintermedius*). Together, these mechanisms facilitate tissue colonization, immune system evasion, chronic infection, and zoonotic transmission—highlighting the need for integrated molecular surveillance and targeted interventions within a One Health framework.

## 4. AMR in Livestock-Associated *Staphylococcus* spp.

AMR in livestock-associated *Staphylococcus* species poses a mounting threat to both veterinary and public health. Recent global surveillance efforts estimate that these bacteria account for 20–30% of MDR isolates in food-producing animals, with the highest prevalence reported in intensive swine and dairy operations [117,118]. Key species implicated include *S. aureus*, *S. pseudintermedius*, and CoNS, which not only contribute to animal infections but also act as reservoirs of transferable resistance genes with zoonotic potential.

### 4.1. Current Detection and Therapeutic Strategies

Diagnostic approaches for livestock-associated *Staphylococcus* spp. integrate conventional phenotypic methods—such as colony morphology, Gram staining, catalase and coagulase testing, and antimicrobial susceptibility assays—with advanced molecular and proteomic tools. PCR assays targeting resistance genes such as *mecA*, *mecC*, and *blaZ* are routinely employed in veterinary diagnostics to identify methicillin resistance [13]. The implementation of WGS and matrix-assisted laser desorption/ionization time-of-flight mass spectrometry (MALDI-TOF MS) has significantly improved strain-level identification, resistance profiling, and epidemiological tracking [119,120].

Therapeutic management of infections remains challenging due to widespread MDR and biofilm formation. Although β-lactams, macrolides, and lincosamides are commonly used as first-line antimicrobials, their clinical efficacy is often undermined by acquired resistance mechanisms. Consequently, therapeutic failures are not uncommon in field settings. Vaccine development represents a promising but still maturing strategy. Autogenous bacterins and recombinant vaccines targeting key virulence factors—such as staphylococcal protein A (SpA), clumping factors A and B (ClfA/B), and leukocidin LukMF′—have demonstrated partial protection, particularly in dairy cattle with a history of recurrent mastitis [121]. However, no broadly effective commercial vaccine is currently available.

### 4.2. Epidemiology and Zoonotic Transmission of MDR Staphylococci

A recent meta-analysis reported that MDR *S. aureus* strains were detected in 27% of isolates from animals, farm personnel, and the environment, commonly resistant to penicillin, ampicillin, tetracycline, chloramphenicol, and ciprofloxacin [122]. Zoonotic transmission is well documented. Guimarães et al. [123] described occupational exposure as a major route. CC398, dominant in pigs and cattle, exhibits host adaptation via the loss of human-specific immune evasion genes (*scn*, *chp*, *sak*), yet retains zoonotic capacity.

### 4.3. Molecular Basis of AMR

MDR staphylococci from livestock often harbor resistance genes including *mecA*, *erm*(B/C), and *tet*(K/M), frequently localized on MGEs. Silva et al. [124] reported the prevalence of *mecA*-positive CC398 isolates in Europe and Asia, co-carrying *tetM*, *ermB*, and *fexA*. Similar findings from South Korea and China support the dominance of this clone [125,126]. Co-selection via metal resistance (*czrC*) and biocide resistance (*qacA/B*), alongside efflux pumps such as NorA and MepA, reinforce persistence and dissemination [127,128].

MRSP is an emerging concern in companion animals. Clones like ST71 and ST258, widespread across continents, exhibit resistance to multiple antibiotic classes. Myrenås et al. [129] found that 96% of Swedish MRSP isolates were MDR, and Teixeira et al. [130] observed similar trends in Brazil. Resistance to disinfectants and robust biofilm formation further complicate management. CoNS species such as *S. chromogenes*, *S. simulans*, and *S. xylosus* are increasingly associated with bovine mastitis. In China, Yang et al. [131] reported 35.2% penicillin and 24.2% tetracycline resistance among CoNS isolates. In Finland, *blaZ*-mediated resistance was widespread in dairy herds [132]. Composite MGEs carrying combinations of resistance genes (e.g., *tet*, *erm*, *blaZ*, *qacA/B*, *copA*) facilitate horizontal gene transfer (HGT) across microbial populations [133,134].

### 4.4. Environmental Dissemination and Reservoirs

Environmental dissemination is a critical yet under-monitored dimension of AMR propagation. Practices such as land application of untreated manure, wastewater discharge, and reuse of contaminated bedding facilitate the release of resistant staphylococci and MGEs into surrounding ecosystems. Studies have documented prolonged persistence of *mecA*- and *qacA/B*-positive strains in adjacent soil and water bodies following manure application [135,136]. MGEs—including plasmids, SCCmec elements, integrative conjugative elements (ICEs), and transposons—enable extensive dissemination of resistance traits. Roadcap et al. [137] detected *blaZ*, *ermC*, *lnuA*, and *aadD* on transposons from bovine isolates, while Namoune et al. [138] reported *mecA*, *tetK*, and *fusC* on MGEs in Algerian strains. Environmental surveillance must therefore be integrated into AMR mitigation strategies.

### 4.5. Foodborne Transmission of AMR Staphylococci

Foodborne exposure is a key route for zoonotic transmission of resistant staphylococci. MRSA and enterotoxin-producing *S. aureus* have been isolated from raw milk, artisanal cheeses, and undercooked meat [139]. In Portugal, 53% of bulk milk samples contained *S. aureus*, with 8.1% harboring *mecA* and 43% expressing enterotoxins *sea*, *seg*, and *sei* [140]. Similarly, Deepak et al. [141] identified high rates of MRSA and biofilm-forming, toxin-positive strains in raw milk. These findings illustrate dual risks: foodborne intoxication and resistance gene transfer via the gut microbiota.

### 4.6. Antimicrobial Usage and Resistance Selection Pressure

Antimicrobial use (AMU) in livestock production exerts intense selective pressure. Prophylactic and metaphylactic antibiotic use in high-density pig and poultry farms fosters MDR strains like MRSA CC398 and MRSP ST71. AMU has been directly correlated with LA-MRSA colonization in animals and handlers [127,136]. In contrast, ruminant systems with stringent AMU policies—such as those in Northern Europe—report lower resistance rates. Surveillance from the European Medicines Agency and FAO confirms that species-specific AMU stewardship significantly reduces AMR burden [117,142]. Commensal *Staphylococcus* species, particularly CoNS, function as silent reservoirs of resistance genes. While often asymptomatic, these organisms can transfer resistance determinants to pathogenic strains under selective pressure [143,144].

### 4.7. Alternative Therapeutics and Preventive Strategies

Non-antibiotic strategies offer promising avenues. Vaccines targeting *S. aureus* (e.g., *ClfA*, *LukMF′*) reduce bacterial load and disease severity [145,146]. Bacteriophage therapy has demonstrated efficacy against biofilm-forming strains, offering species specificity and minimal microbiota disruption [147]. Antivirulence agents—including quorum sensing inhibitors and immunomodulators—also show potential [148]. Probiotics such as *Lactobacillus plantarum* CM49 may displace *S. aureus* and restore microbiome balance, reducing mastitis incidence [149].

### 4.8. Surveillance Gaps and One Health Priorities

Despite regional initiatives—such as ESVAC (Europe), NARMS (USA), and China’s AMR surveillance programs—substantial gaps persist in the global monitoring of livestock-associated *Staphylococcus* spp. Most surveillance systems disproportionately focus on *S. aureus*, with limited attention to CoNS and environmental reservoirs [142]. Routine implementation of WGS and MGE profiling remains limited, particularly in low- and middle-income countries (LMICs), and the lack of interoperable data platforms hinders cross-sectoral integration. Environmental reservoirs of AMR—including manure, wastewater, and agricultural soils—remain significantly under-characterized, despite growing evidence of their role in the long-distance dissemination of resistant *Staphylococcus* spp. and associated resistance genes [117]. The absence of harmonized surveillance frameworks and standardized metadata protocols continues to impede progress in One Health coordination.

To overcome these challenges, a globally integrated One Health surveillance infrastructure is urgently needed—built upon standardized sequencing methodologies, real-time data sharing, and international regulatory collaboration. AMU stewardship must extend beyond clinical settings to encompass veterinary, agricultural, and environmental domains, with policies tailored to the specific risks of different animal species and production systems. Future research priorities should include predictive modeling, resistome mapping, and functional metagenomics to proactively detect and track the emergence of high-risk clones and resistance networks.

Robust antimicrobial stewardship programs in veterinary contexts—aligned with One Health principles—are essential for mitigating the selection, amplification, and environmental spread of MDR clones, as well as minimizing zoonotic transmission risks. Beyond MDR strains, *Staphylococcus* spp. displaying extensive drug resistance (XDR) and even pan-drug resistance (PDR) have been sporadically reported, posing serious challenges for both veterinary and human health interventions [13,150]. Looking ahead, the emphasis must be on strengthening real-time genomic surveillance, expanding functional metagenomics, and implementing AI-driven predictive analytics to anticipate resistance trends before they become endemic. However, while AI and ML offer promising avenues for predicting AMR emergence, resistance gene dissemination, and outbreak forecasting, their application in veterinary AMR surveillance remains in its early stages. Current AI/ML models often rely on incomplete or biased training datasets, suffer from variability in metadata quality, and lack external validation across diverse host species, production systems, and geographic regions [151,152]. These limitations hinder the generalizability and real-world applicability of predictive tools. Therefore, although AI holds great potential for enhancing AMR risk assessment, its integration into surveillance programs must proceed cautiously—anchored in algorithmic transparency, interpretability, and rigorous empirical validation [151,153]. Ensuring that predictive models are both biologically meaningful and contextually relevant is essential for their responsible deployment in One Health strategies.

**Figure 4:**
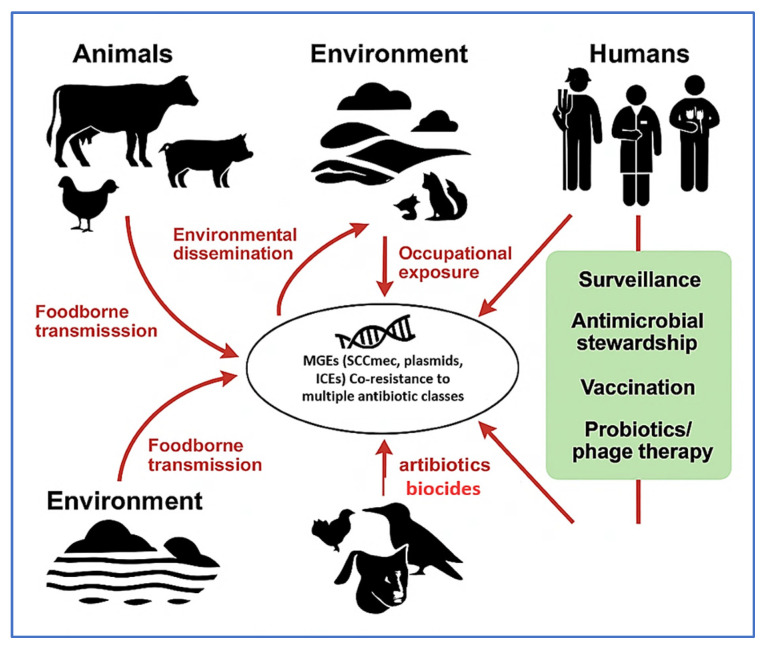
Transmission dynamics and antimicrobial resistance (AMR) dissemination pathways of livestock-associated *Staphylococcus* spp. under the One Health framework. The diagram illustrates the interconnected roles of animals, the environment, and humans in the transmission of AMR *Staphylococcus* species. Mobile genetic elements (MGEs), including Staphylococcal Cassette Chromosome (SCCmec), plasmids, and Integrative and Conjugative Elements (ICEs), act as key vectors for resistance gene dissemination and co-selection. Red arrows indicate major transmission routes (e.g., foodborne exposure, occupational contact, and environmental dissemination), while green highlights denote strategic control measures such as surveillance, antimicrobial stewardship, vaccination, and probiotic/phage-based interventions. This figure was created using a combination of BioRender.com (via a private academic free trial account) and Microsoft PowerPoint (Microsoft 365), both of which permit use in open-access scientific publications.

Figure 4 conceptually illustrates the ecological dissemination routes of resistant *Staphylococcus* species across animal, human, and environmental reservoirs. These include foodborne transmission via contaminated animal products, occupational exposure, and environmental leakage through wastewater runoff and wildlife interactions. Migratory birds, in particular, serve as long-range vectors for ARGs, with resistant *Staphylococcus* strains isolated from gulls, pigeons, and waterfowl near livestock facilities and urban environments [154]. The diagram also highlights key intervention points—such as vaccination, stewardship, and targeted diagnostics—within a One Health surveillance framework.

**Figure 5:**
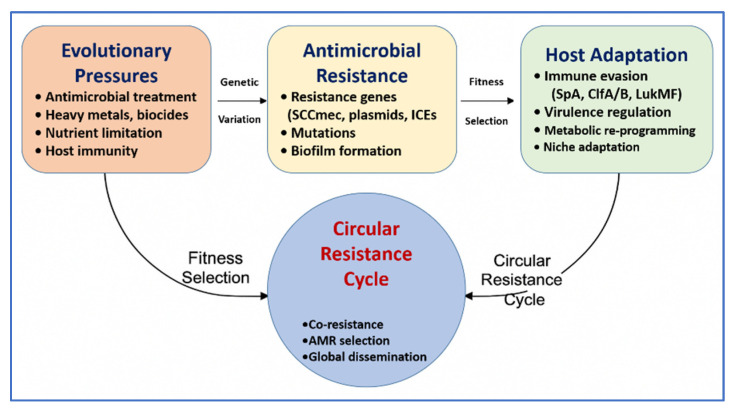
Circular resistance cycle driving the evolution and host adaptation of livestock-associated *Staphylococcus* species. This schematic illustrates the core mechanisms contributing to antimicrobial resistance (AMR) persistence and spread: evolutionary pressures (e.g., antimicrobials, host immunity), genetic variation, antimicrobial resistance traits (e.g., mobile genetic elements (MGEs), mutations), and host adaptation (e.g., virulence regulation, immune evasion). These factors interact via fitness selection to drive the cycle of co-resistance and global dissemination. This figure was created using Microsoft PowerPoint (Microsoft 365), which is permitted for use in open-access scientific publications.

To complement this, Figure 5 depicts the circular resistance cycle, showing how evolutionary pressures—including AMU, host immunity, and environmental stressors—drive genetic diversification, antimicrobial resistance, and host adaptation. This feedback loop facilitates fitness selection, co-resistance, and the emergence of dominant MDR clones. Together, Figure 4 and Figure 5 emphasize the interconnected ecological and molecular dynamics that must be addressed to control the spread of resistance. Table 2 further complements these insights by comparing the operational characteristics, performance, and limitations of AMR detection tools across farm, processing, and clinical settings. The combined interpretation of Figure 4 and Figure 5, along with Table 2, provides a strategic foundation for advancing evidence-based surveillance and intervention strategies in livestock-associated *Staphylococcus* spp.

Table 2 provides a comparative overview of major livestock-associated *Staphylococcus* species, detailing their primary animal hosts, key AMR genes, phenotypic resistance profiles, zoonotic potential, and supporting literature. Notably, *S. aureus* CC398 dominates in swine and cattle populations, frequently carrying *mecA*, *tetM*, and *ermB*, and poses a significant zoonotic threat, particularly through occupational exposure. *S. pseudintermedius* lineages such as ST71 and ST258, prevalent in canine infections, exhibit broad multidrug resistance and have been implicated in human–pet transmission events. Coagulase-negative *Staphylococcus* (CoNS) species—including *S. chromogenes*, *S. simulans*, and *S. xylosus*—once considered minor mastitis pathogens, are now increasingly recognized for their multidrug resistance and environmental persistence. This tabulated synthesis reinforces the necessity of species-level surveillance and resistance monitoring within veterinary microbiology and supports the broader One Health approach to AMR containment.

## 5. Genomic Surveillance and Data Harmonization Gaps

Global AMR monitoring remains uneven, with a skewed focus on *S. aureus*. Despite significant advancements in AMR surveillance, global genomic monitoring of livestock-associated *Staphylococcus* spp. remains uneven and disproportionately focused on *S. aureus*, while CoNS are underrepresented despite their increasing role as resistance gene reservoirs. WGS has proven invaluable in identifying dominant MDR clones, MGEs, and interspecies transmission pathways. For instance, Monecke et al. [12] used WGS to characterize MRSA CC398 isolates from pigs and farm workers in Germany, revealing high clonal diversity and multiple SCCmec variants associated with zoonotic transmission. Livestock production environments facilitate the spread of MDR clones. Recent surveillance efforts have further confirmed the global dissemination of livestock-associated *S. aureus* ST398 and ST541 clones, particularly in swine production systems, where extensive antimicrobial use facilitates the acquisition of resistance determinants. Sun et al. [155] reported a high prevalence of *tet(M)* and *erm(B)* genes among ST398 isolates from Chinese pig farms, with genomic evidence linking these traits to MGEs and environmental co-selection pressures.

Similarly, Lee et al. [60] similarly documented MRSA CC398 and ST541 in Korean swine, identifying *tet(M)*, *erm(B)*, and the linezolid-resistance gene *cfr* in isolates from pigs, farm environments, and workers, underscoring both zoonotic transmission and MDR phenotypes. WGS reveals significant resistance and virulence potential in CoNS. A genomics-based investigation by Sharma et al. [156] further substantiated the pathogenic potential of CoNS. Through WGS of 22 NAS strains from clinical bovine mastitis cases, the study revealed that species like *S. chromogenes*, *S. haemolyticus*, and *S. sciuri* not only harbored multiple AMR determinants—such as *tet(K)*, *erm(C)*, and *aac(6′)-Ie-aph(2″)-Ia*—but also displayed virulence profiles and biofilm formation capabilities comparable to those of *S. aureus*. Many of these genes were embedded within MGEs, particularly plasmids and genomic islands, highlighting their capacity for horizontal gene transfer and persistence under antimicrobial pressure. However, surveillance of MGEs remains fragmented, limiting insight into how resistance spreads across niches. Persson Waller et al. [157] identified ICEs carrying *blaZ*, *qacA*, and aminoglycoside resistance genes in *S. chromogenes* and *S. simulans* from Swedish dairies, often co-located with virulence regulators (*agr*, *sarA*). Likewise, Zhang et al. [138] detected *SCCmec*-like cassettes and IS431-bearing transposons in CoNS from Chinese mastitis cases, spanning *S. sciuri*, *S. hominis*, and *S. capitis*.

In a large-scale resistome profiling study, Zhang et al. [158] investigated CoNS isolated from clinical bovine mastitis cases in China. The isolates—spanning species such as *S. sciuri*, *S. hominis*, and *S. capitis*—harbored an array of resistance genes including *blaZ*, *ermC*, and *tetK*, many of which were integrated within SCCmec-like chromosomal cassettes. The detection of composite transposons and insertion sequence IS431 further supported the high mobility and interspecies transfer potential of these resistance elements. The urgency to standardize MGE surveillance in veterinary microbiology has increased in light of the growing complexity of resistomes in livestock-associated *Staphylococcus* spp. There is a critical need to harmonize MGE annotation frameworks. A genomic investigation by Becker et al. [159] revealed substantial heterogeneity in *SCCmec* elements and plasmid architectures among livestock-associated *S. aureus* isolates from European farms. Their findings underscored the lack of consistency in MGE annotation practices across laboratories, which hinders effective comparative surveillance. Accordingly, the authors advocated for the development of centralized platforms to harmonize *SCCmec* classification and plasmid typing protocols. In parallel, Lee et al. [60] conducted a global genomic survey of MRSA isolates across more than 30 countries, identifying significant discrepancies in SCCmec annotation and metadata reporting. Their proposed solution—a community-curated reference framework—aims to facilitate inter-laboratory comparability and strengthen global resistome tracking.

Evidence of interspecies resistance gene transfer is growing. Gao et al. [160] reported on the horizontal dissemination of *cfr*-carrying conjugative plasmids across multiple *Staphylococcus* species originating from both animal and human sources. Their data demonstrated interspecies gene flow involving critical resistance determinants such as *cfr*, *erm(B)*, and *fexA*, highlighting the dual zoonotic and environmental dimensions of AMR propagation. Collectively, these studies illustrate that CoNS are not passive commensals but active participants in the evolution and spread of AMR within livestock ecosystems. Their genomic plasticity, ecological versatility, and capacity to acquire and disseminate MGEs place them at the forefront of emerging AMR threats.

While WGS has advanced the detection of AMR genes, short-read technologies often fail to resolve complex MGE architectures, particularly in CoNS. Integrating long-read sequencing platforms, such as Oxford Nanopore and PacBio, enables accurate reconstruction of plasmids, transposons, and ICEs, which are frequently misassembled in draft genomes [161]. Additionally, metagenomic sequencing of livestock environments can uncover uncultured reservoirs of AMR, offering a more comprehensive view of resistance gene flow in farm ecosystems [162]. Together, these approaches are essential for closing the current gaps in MGE surveillance and resistome tracking within One Health frameworks.

Addressing this challenge requires a shift toward integrated, One Health surveillance systems that incorporate long-read sequencing, metagenomic analyses, and comprehensive mobilome profiling. Such frameworks are essential for timely detection, risk assessment, and containment of resistance dissemination across animal and human health domains. To complement these findings, Table 3 summarizes representative studies highlighting MGEs, resistance determinants, and their mechanisms of dissemination across livestock-associated *Staphylococcus* spp. in diverse geographic settings.

To address the growing complexity of AMR in livestock-associated *Staphylococcus* spp., global AMR governance must prioritize enhanced data integration, capacity building, and technology transfer. Expanding One Health genomic surveillance beyond *S. aureus* to include CoNS, and incorporating mobilome data into international repositories—such as the WHO Global Antimicrobial Resistance Surveillance System (GLASS) and the NCBI Pathogen Detection platform—are vital for closing current surveillance gaps [164]. Investment in real-time sequencing infrastructure, particularly in low- and middle-income countries, remains crucial to ensure equitable access to modern resistome monitoring tools [165]. Emerging digital tools—such as AI-driven resistance prediction and cloud-based platforms for MGE annotation—are reshaping AMR surveillance. ML models have shown high accuracy in predicting resistance phenotypes in *Staphylococcus* spp. [166], while deep learning approaches enable rapid resistome profiling in complex samples [167]. Platforms like MobileElementFinder support automated MGE detection from WGS data, enhancing integration into One Health frameworks [168].

To future-proof genomic surveillance of AMR in livestock-associated *Staphylococcus* spp., it is critical to adopt a more equitable, collaborative, and innovation-driven approach. Investment in mobile sequencing platforms—such as Oxford Nanopore MinION—offers scalable, field-deployable options for real-time AMR monitoring, particularly in under-resourced regions [169]. Public–private partnerships and open-source bioinformatics tools like ARIBA and ResFinder have also enabled more accessible resistome analytics, reducing dependency on high-cost proprietary pipelines [170,171]. However, sustainability challenges remain. Long-term success hinges on establishing regional AMR genomics hubs, fostering local expertise, and implementing FAIR (Findable, Accessible, Interoperable, and Reusable) data-sharing principles across One Health sectors [172,173]. Only through coordinated global action and capacity building can genomic surveillance fully support early warning systems and targeted interventions against the spread of resistance across the food-animal–human interface.

## 6. Future Perspectives and Research Directions

The persistence and global spread of MDR *Staphylococcus* species in livestock represents a critical challenge at the intersection of veterinary medicine, food safety, and public health. Although substantial progress has been achieved in characterizing AMR and virulence traits through WGS, significant knowledge gaps remain in the mechanistic understanding, predictive modeling, and translational application of these insights. Future research must strategically transition toward integrative, high-resolution approaches that enable early detection, targeted intervention, and long-term containment of zoonotic and environmentally persistent *Staphylococcus* clones.

### 6.1. Functional Genomics and Molecular Dissection of Virulence

Despite the rapid expansion of genomic databases, the functional roles of many resistance and virulence genes in *Staphylococcus* spp. remain poorly understood, particularly within the host–pathogen interface in livestock. High-throughput functional genomics platforms—such as CRISPR interference (CRISPRi) and transposon insertion sequencing (Tn-seq)—have emerged as transformative tools for dissecting gene essentiality and regulatory networks. Recent genome-wide CRISPRi screens in *S. aureus* have uncovered previously uncharacterized genes involved in antibiotic susceptibility to lipoglycopeptides such as dalbavancin, underscoring CRISPRi’s capacity to probe essential and conditionally essential loci inaccessible by traditional mutagenesis approaches [174]. Moreover, integrative applications of CRISPRi and Tn-seq have validated key genetic determinants required for macrophage invasion and intracellular survival [175]. Complementary in vivo Tn-seq analyses have also delineated metabolic and virulence gene networks essential for *S. aureus* persistence in infection models such as osteomyelitis [176]. Expanding the use of these functional genomics approaches across diverse livestock-associated strains—including *S. pseudintermedius* and CoNS—will be instrumental in uncovering novel therapeutic targets and elucidating the contribution of MGEs to host adaptation and pathogenicity.

### 6.2. Environmental Metagenomics and Resistome Mapping

Environmental compartments—including soil, surface water, bioaerosols, and manure-amended fields—represent critical yet under-monitored reservoirs for AMR genes originating from livestock production systems. These environments often harbor diverse resistance determinants such as *mecA*, *tet(M)*, *erm(B)*, and *sul1*, many of which are embedded within MGEs that facilitate horizontal gene transfer and long-term environmental persistence [177,178]. Manure runoff and wastewater effluents from pig, poultry, and dairy farms have been shown to contaminate adjacent ecosystems with a high load of AMR genes and human-associated pathogens. Fang et al. [177] demonstrated that ARGs and MDR bacteria—including *Escherichia coli*, *Enterococcus* spp., and *Staphylococcus* spp.—were disseminated from a pig feedlot into downstream water bodies and agricultural soils. Similarly, Guo et al. [178] performed metagenomic analyses of municipal and agricultural wastewater, revealing widespread localization of ARGs on plasmids, ICEs, and class 1 integrons, with a high potential for cross-species gene transfer.

Airborne transmission of ARGs is increasingly recognized as a nontraditional yet significant route of AMR dissemination from livestock operations. Recent metagenomic analyses by Xu et al. [179] revealed that bioaerosols collected from the interior and periphery of poultry houses harbored a diverse set of ARGs, including *blaTEM*, *tet(Q)*, *erm(B)*, and *sul1*, many of which were associated with MGEs. These airborne particles contained viable bacteria with the potential for horizontal gene transfer, underscoring the role of farm-generated aerosols in environmental and occupational AMR exposure. In parallel, agricultural soils subjected to repeated applications of livestock manure act as long-term reservoirs of ARGs, especially under co-selection pressure from antimicrobials and heavy metals. Wang et al. [180] showed that the combined presence of tetracycline and copper in manured soils significantly enhanced both the abundance and diversity of ARGs—including *tet(G)*, *tet(B)*, *tet(Q)*, *sul1*, *sul2*, and *intI1*—by facilitating the proliferation of MGEs. These findings emphasize the ecological synergy between antibiotics and co-contaminants in shaping resistome persistence and mobility in terrestrial ecosystems. Collectively, these studies highlight the urgent need to include air and soil compartments in One Health-based AMR surveillance frameworks.

To comprehensively capture the environmental resistome, future surveillance systems should integrate shotgun metagenomics with long-read sequencing technologies (e.g., PacBio, Menlo Park, CA, USA; Oxford Nanopore, Oxford, UK). These tools provide enhanced resolution of complex MGEs, co-localized resistance and virulence loci, and complete plasmid architectures—offering unprecedented insights into AMR dynamics beyond cultured isolates. As Fournier et al. [181] highlighted, environmental DNA (eDNA) and metagenomics are indispensable for tracing resistance genes across clinical, veterinary, and environmental interfaces, and for informing risk assessments in One Health frameworks.

### 6.3. Multi-Omics Approaches to Host Adaptation and Niche Specialization

The successful adaptation of *Staphylococcus* species to specific livestock hosts is driven by a multifaceted interplay of genomic traits, immune system pressures, and environmental nutrient constraints. Integrative multi-omics approaches—including pan-genomics, transcriptomics, proteomics, and metabolomics—have proven indispensable for elucidating the molecular underpinnings of host specificity, ecological fitness, and long-term colonization. Pan-genomic and transcriptomic analyses have uncovered lineage-specific genes and regulatory networks associated with enhanced virulence and host adaptation. Capra et al. [182] demonstrated that *S. aureus* isolates from high-prevalence bovine herds exhibited upregulation of genes linked to adhesion, biofilm formation, and iron acquisition, highlighting the evolutionary selection of traits that promote persistence in the mammary gland. These findings support the hypothesis that accessory genome elements play a central role in shaping strain-specific host tropism.

Complementary proteomic studies have further dissected host–pathogen interactions at the protein level. Eckersall et al. [183] performed comparative proteomics on bovine *S. aureus* isolates and found significant upregulation of proteins involved in lactose metabolism, iron transport, and oxidative stress responses during intramammary infections. Such adaptive metabolic reprogramming reflects the bacterium’s tailored fitness in the milk-rich, immunologically active mammary environment, and provides candidate biomarkers for mastitis diagnostics and vaccine design. Expanding beyond bovine systems, Sawhney et al. [184] employed comparative genomics across canine and human *S. pseudintermedius* isolates and identified host-specific differences in CRISPR-Cas systems, prophage profiles, and AMR determinants. Their study revealed that niche specialization is tightly linked to the acquisition and regulation of MGEs and immune evasion strategies, underscoring the dynamic genome plasticity of staphylococci in response to host pressures.

In addition, metabolomics is emerging as a valuable layer of the multi-omics framework. Recent milk metabolome profiling by Reuben and Torres [185] revealed distinct signatures of fatty acid derivatives, amino acid metabolism, and proinflammatory mediators in subclinical mastitis cases, corresponding with specific staphylococcal colonization patterns. These host metabolic responses provide insights into pathophysiological changes during infection and open new avenues for precision diagnostics. Together, multi-omics platforms offer a systems-level perspective on how staphylococci evolve and persist in specific livestock hosts. By linking genomic potential to expressed phenotypes and host metabolic context, these approaches can identify conserved molecular signatures—such as iron acquisition operons, surface adhesins, and detoxification enzymes—that represent promising targets for next-generation diagnostics, therapeutic interventions, and subunit vaccines tailored to species-specific pathogens.

### 6.4. Translational Validation of Alternative Therapeutics

As the global efficacy of conventional antibiotics continues to decline, alternative antimicrobial strategies are being actively explored for application in livestock-associated *Staphylococcus* infections. Among these, nanoparticle-based antimicrobials, bacteriophage therapy, and probiotic interventions have shown promising preclinical outcomes, yet their translational validation remains limited. Zinc oxide nanoparticles (ZnO-NPs) have demonstrated potent activity against MDR and biofilm-forming *S. aureus* strains. In a recent study, Abdelghafar et al. [186] reported that ZnO-NPs significantly disrupted mature biofilms and reduced bacterial burden in a murine wound infection model. This antimicrobial effect was accompanied by downregulation of biofilm-associated genes and suppression of virulence expression. Building on this, Hao et al. [187] demonstrated that ZnO-NPs could synergize with conventional antibiotics, such as oxacillin and vancomycin, enhancing their efficacy while attenuating hemolysin production and biofilm biomass. These findings support the therapeutic potential of ZnO-NPs as adjuncts or alternatives to traditional antibiotics in veterinary infections caused by *Staphylococcus* spp. However, the therapeutic use of zinc-based compounds should be approached with caution. The *czrC* gene, often co-located with *mecA* on SCCmec elements—particularly types V and IX—confers resistance to zinc and has been implicated in the co-selection of LA-MRSA under zinc oxide exposure. This genetic linkage enhances the persistence of MRSA strains in zinc-supplemented environments, prompting regulatory efforts to phase out zinc oxide in animal feed [188].

Bacteriophage therapy is also gaining renewed interest. A systematic evaluation by Nale et al. [189] highlighted the efficacy of phage cocktails targeting *S. aureus* and *S. pseudintermedius* in both in vitro and in vivo mastitis models. Phage application reduced bacterial counts, biofilm viability, and inflammatory markers in bovine mammary tissues. However, phage therapy in agriculture still faces significant translational hurdles, including regulatory approval, narrow host specificity, and lack of standardized delivery protocols for field use. Probiotics, particularly *Lactobacillus plantarum* (*L. plantarum*), offer another biologically sustainable approach. Li et al. [190] engineered *L. plantarum* strains capable of sensing *S. aureus* quorum-sensing signals and secreting lysostaphin, thereby directly inhibiting pathogen growth. Moreover, field trials involving *L. plantarum* CM49 in dairy cattle demonstrated enhanced udder health, reduced somatic cell counts, and decreased colonization by *Staphylococcus* spp., suggesting immunomodulatory and competitive exclusion effects. Despite these promising findings, most studies remain confined to laboratory or small-animal models. To establish their practical utility, these alternative therapeutics must undergo rigorous large-scale clinical trials in livestock environments. Such trials should evaluate not only antimicrobial efficacy but also delivery feasibility, host microbiota impacts, safety profiles, and cost-effectiveness within real-world farm conditions.

### 6.5. Artificial Intelligence (AI) and Predictive Surveillance Systems

AI and ML are emerging as transformative tools in the surveillance, prediction, and management of AMR. By leveraging large-scale genomic and epidemiological datasets, AI-driven platforms offer unprecedented capabilities in detecting resistance phenotypes, tracking MGEs, and forecasting AMR trends in both clinical and agricultural settings. Recent studies have demonstrated the feasibility of ML-based models in accurately predicting AMR phenotypes in *S. aureus*. Wang et al. [191] trained ML algorithms—including support vector machines and gradient boosting classifiers—on genome-wide k-mer data from *S. aureus* isolates. Their models achieved high predictive accuracy for minimum inhibitory concentrations of key antibiotics, such as oxacillin and erythromycin, demonstrating the potential for rapid, culture-independent resistance profiling using WGS data. Beyond resistance prediction, AI systems are being applied to epidemiological modeling and outbreak risk assessment. Lin et al. [192] utilized a MaxEnt ecological niche modeling approach, integrating electronic health records and environmental features to identify geographic hotspots of community-onset *S. aureus* infections. Their model achieved strong discriminatory power (AUC: 0.77–0.84), highlighting the utility of AI in guiding spatially targeted AMR interventions.

Furthermore, AI-assisted bioinformatics tools have advanced the detection and classification of MGEs and resistance determinants from metagenomic datasets. For instance, Khabaz et al. [193] developed a deep learning framework to identify antimicrobial peptides with anti-*Staphylococcus* activity, while modern resistome databases such as the Comprehensive Antibiotic Resistance Database (CARD) now incorporate ML-enhanced modules for predicting novel resistance gene candidates. These developments enhance both sensitivity and precision in AMR surveillance and functional annotation. Despite these advancements, the full translational potential of AI in veterinary AMR surveillance depends on the standardization of genomic metadata, improved data quality, and international collaboration for open data sharing. Establishing interoperable data pipelines and ethical frameworks will be critical to ensure reproducibility, transparency, and scalability of AI tools in livestock health monitoring and One Health surveillance networks.

**Figure 6:**
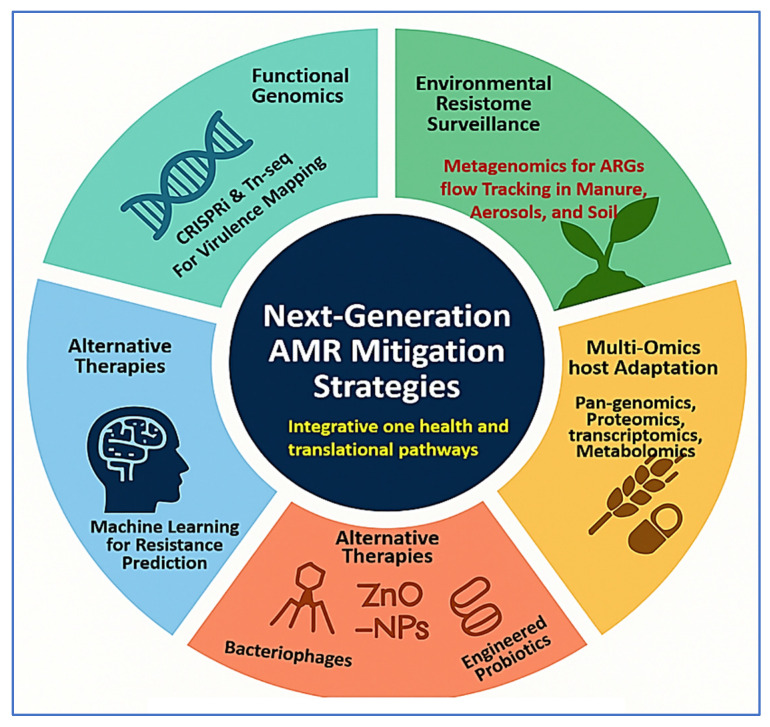
Conceptual diagram illustrating next-generation antimicrobial resistance (AMR) mitigation strategies for livestock-associated *Staphylococcus* species. The figure presents an integrated One Health framework involving five core research domains: functional genomics, environmental resistome surveillance, multi-omics host adaptation, AI-based prediction tools, and alternative therapeutics. This figure was created using a combination of BioRender.com (via a private academic free trial account) and Microsoft PowerPoint (Microsoft 365), both of which permit use in open-access scientific publications.

To visually encapsulate the integrative directions outlined in Section 6, Figure 6 summarizes the five major pillars of future AMR mitigation in livestock-associated Staphylococcus. These include functional genomics (e.g., CRISPRi, Tn-seq), metagenomics-based resistome mapping, host-adaptation insights via pan-omics platforms, AI-driven surveillance systems, and innovative alternative therapies (e.g., ZnO-NPs, bacteriophages, engineered probiotics). This conceptual map reflects a translational research pathway grounded in the One Health paradigm, aimed at sustainable AMR containment across agricultural ecosystems.

## 7. Conclusions

The emergence and persistence of MDR *Staphylococcus* spp. in livestock present an escalating challenge at the crossroads of veterinary medicine, public health, and food safety. Despite the extensive application of WGS and molecular surveillance, critical gaps remain in translating these insights into actionable mitigation strategies. This review underscores the importance of a systems-level approach—integrating functional genomics, environmental metagenomics, multi-omics profiling, and AI—to dissect the adaptive mechanisms, ecological drivers, and epidemiological dynamics of AMR in livestock-associated *Staphylococcus*. Future research must prioritize the functional validation of resistance and virulence determinants using high-throughput genomic tools such as CRISPRi and Tn-seq, especially in underexplored species like *S. pseudintermedius* and coagulase-negative staphylococci. The incorporation of environmental compartments—air, water, soil, and manure—into AMR monitoring frameworks is equally essential to capture the full resistome and prevent cross-sector dissemination. Moreover, multi-omics strategies will be pivotal in elucidating host specificity, niche adaptation, and the metabolic reprogramming that underpins chronic infections and zoonotic potential. Complementing these efforts, AI-powered prediction models and metagenomic analytics hold promise for enhancing real-time surveillance and informing tailored antimicrobial stewardship. The translational pipeline must also be strengthened to validate alternative therapeutics—such as nanoparticles, bacteriophages, and engineered probiotics—through large-scale clinical trials in livestock environments. Collectively, addressing the AMR burden posed by *Staphylococcus* spp. in animal agriculture requires a forward-looking, interdisciplinary paradigm rooted in the One Health approach. Only through collaborative, data-driven innovation can we develop sustainable interventions to protect animal welfare, safeguard food chains, and curb the global spread of AMR.

## Figures and Tables

**Table 1:** Genomic mechanisms of host adaptation in livestock-associated *Staphylococcus* spp.

Mechanism	Genetic Elements Involved	*Staphylococcus* Species	Host Adaptation Outcome	References
Adhesion to host tissues	*clfB*, *fnbB*, *cna*	*S. aureus*	Enhanced binding to bovine mammary epithelial cells	[84]
Immune evasion	Loss of *scn*, *chp*, *sak*	*S. aureus* CC398	Adaptation to livestock hosts with reduced human immune evasion	[78]
Metabolic adaptation	lac operon, iron uptake genes	*S. aureus*, *S. chromogenes*	Improved growth in milk and low-iron environments	[42,45]
Toxin production	*lukMF′*, *ExhA-D*	*S. aureus*, *S. hyicus*	Host-specific cytotoxicity and epithelial damage	[85]
AMR	SCCmec, *tetM*, *ermB*	*S. aureus*, *S. pseudintermedius*	Survival under antibiotic pressure in farm environments	[13,86]
Biofilm formation	*icaABCD* operon	*S. pseudintermedius*, *S. chromogenes*	Persistence on mucosal surfaces and resistance to treatment	[87,88]

AMR, antimicrobial resistance; SCCmec, staphylococcal cassette chromosome *mec*; *clfB*, clumping factor B; *fnbB*, fibronectin-binding protein B; *cna*, collagen adhesin; *scn*, staphylococcal complement inhibitor; *chp*, chemotaxis inhibitory protein; *sak*, staphylokinase; lukMF′, leukocidin MF′; ExhA-D, exfoliative toxins A to D; *tetM*, tetracycline resistance gene; *ermB*, macrolide resistance gene; icaABCD, intercellular adhesion operon.

**Table 2:** Summary of livestock-associated *Staphylococcus* spp. with references.

Staphylococcus Species	Primary Hosts	Key AMR Genes	AMR Phenotype	Zoonotic Potential	Key References
*S. aureus* (CC398)	Pigs, Cattle	*mecA*, *tetM*, *ermB*, *fexA*, *cfr*	MDR, β-lactam, macrolide, tetracycline, phenicol resistance	High (occupational exposure)	[60,124,126]
*S. pseudintermedius* (ST71, ST258)	Dogs	*blaZ*, *ermB*, *tetM*, *aac(6′)-aph(2″)*, *qacJ*	MDR, chlorhexidine resistance, strong biofilm formation	Confirmed (human–pet transmission)	[64,129]
*S. chromogenes*	Dairy Cattle	*blaZ*, *tetK*, *aac(6′)-aph(2″)*	Penicillin, tetracycline resistance	Possible (environmental exposure)	[131]
*S. simulans*	Dairy Cattle	*blaZ*, *tetK*	Penicillin resistance	Low (commensal, emerging)	[132]
*S. xylosus*	Dairy Cattle	*blaZ*	Mild β-lactam resistance	Low (commensal)	[132]

AMR: Antimicrobial Resistance; MDR: Multidrug Resistance; β-lactam: Beta-lactam antibiotics including penicillin and cephalosporins; SCCmec: Staphylococcal Cassette Chromosome mec; aac(6′)-aph(2″): Aminoglycoside resistance gene encoding bifunctional enzyme.

**Table 3:** Overview of representative studies describing MGEs, AMR genes, and transfer mechanisms in livestock-associated *Staphylococcus* species across different countries. Data include key species, detected resistance determinants, associated MGEs, and contextual drivers such as zoonotic or environmental selection.

Study	Country	Species/Clones	Key AMR Genes Detected	MGEs Identified	Key Findings
Monecke et al. [12]	Germany	MRSA CC398	*tet(M)*, *erm(B)*, *blaZ*	SCCmec (multiple types)	High clonal diversity; zoonotic MRSA
Li et al. [163]	China	*S. aureus* ST398	*tet(M)*, *erm(B)*	Plasmids, transposons	Environmental and antimicrobial co-selection pressures
Lee et al. [60]	South Korea	MRSA ST398, ST541	*cfr*, *erm(B)*, *tet(M)*	SCCmec, plasmids	Zoonotic transmission, MDR phenotypes
Sharma et al. [156]	India	NAS strains (*S. chromogenes*, *S. sciuri*)	*tet(K)*, *erm(C)*, *aac(6′)-Ie-aph(2″)-Ia*	Plasmids, genomic islands	Virulence and resistance profiles akin to S. aureus
Waller et al. [157]	Sweden	*S. chromogenes*, *S. simulans*	*blaZ*, *qacA*, aminoglycoside genes	ICEs, agr, sarA	Co-localization of resistance and virulence genes
Zhang et al. [158]	China	CoNS from mastitis (*S. sciuri*, *S. hominis*)	*blaZ*, *ermC*, *tetK*	SCCmec-like cassettes, IS431	High mobility and interspecies transmission potential
Becker et al. [159]	Europe	Livestock-associated *S. aureus*	*mecA*, *tetK*, *erm(B)*	Diverse SCCmec, plasmid types	Need for standardized MGE annotation
Gao et al. [160]	China	*S. aureus*, *S. sciuri*, *S. capitis*	*cfr*, *erm(B)*, *fexA*	Conjugative plasmids	Cross-species transfer of multidrug resistance

AMR, antimicrobial resistance; MRSA, methicillin-resistant *Staphylococcus aureus*; NAS, non-aureus staphylococci; CoNS, coagulase-negative staphylococci; SCCmec, staphylococcal cassette chromosome mec; ICEs, integrative and conjugative elements; IS431, insertion sequence 431; MGEs, mobile genetic elements; MDR, multidrug resistance.

## Data Availability

Not applicable. No new data were created or analyzed in this study.
